# The role of the radiologist in the assessment of thoracic changes after radiotherapy

**DOI:** 10.1590/0100-3984.2020.0070

**Published:** 2021

**Authors:** Sabrina de Mello Ando, Eduardo Kaiser Ururahy Nunes Fonseca, Julliana dos Santos Frassei, Lucas de Pádua Gomes de Farias, Yuri Costa Sarno Neves, Rodrigo Caruso Chate, Márcio Valente Yamada Sawamura

**Affiliations:** 1 Instituto de Radiologia do Hospital das Clínicas da Faculdade de Medicina da Universidade de São Paulo (InRad/HC-FMUSP), São Paulo, SP, Brazil.; 2 Departamento de Imagem - Hospital Israelita Albert Einstein, São Paulo, SP, Brazil.

**Keywords:** Radiotherapy, Tomography, X-ray computed, Magnetic resonance imaging, Diagnostic imaging, Thoracic neoplasms, Radioterapia, Tomografia computadorizada, Ressonância magnética, Diagnóstico por imagem, Neoplasias torácicas

## Abstract

Radiotherapy plays a central role in the palliative and curative treatment of neoplasms of the chest wall or intrathoracic structures. However, despite technical advances, radiotherapy can alter previously normal organs and tissues, those alterations presenting as various types of imaging findings. Post-radiation alterations must be promptly recognized by radiologists, in order to avoid confusion between complications of radiotherapy and the recurrence of a tumor. This pictorial essay aims to illustrate different thoracic changes after radiotherapy.

## Abstract (pt)

A radioterapia tem papel fundamental no tratamento curativo ou paliativo de neoplasias da parede torácica ou intratorácicas. No entanto, mesmo com o avanço das técnicas, órgãos e tecidos vizinhos podem ser acometidos e apresentar alterações que devem ser reconhecidas pelos radiologistas, de maneira a evitar confusão com recidiva das lesões inicialmente tratadas, ou mesmo com outras complicações do tratamento oncológico. O objetivo deste ensaio iconográfico é ilustrar as diferentes consequências torácicas da radioterapia.

## INTRODUCTION

Radiotherapy can be used, with curative, palliative, or adjuvant intent, in the treatment of malignant tumors of the chest wall or intrathoracic structures, being used in isolation or in conjunction with surgery or chemotherapy^(1,2)^. In addition to conventional radiotherapy, other techniques are emerging, such as conformational, modulated, and stereotactic radiotherapy, as well as proton beam therapy, the objective being to increase the radiation dose in the tumor while reducing the dose in healthy tissues. Even when the newest techniques are employed, adjacent organs and tissues can undergo radiation-induced changes. This article aims to illustrate various thoracic complications after radiotherapy^(3)^.

## LUNGS

Radiation-induced lung injury has three sequential pathological phases: an acute exudative phase; an organizing, or proliferative, phase; and a chronic fibrotic phase. The acute exudative phase typically manifests 4-12 weeks after the end of radiotherapy, during which time there can be repair. Some of the changes may persist, progressing to the proliferative phase, which begins after 3-9 months, or to the fibrosis phase, which typically begins between 9 months and 2 years after the exposure^(1,4)^.

A number of factors influence the degree of lung injury, mainly the volume of the irradiated lung, the total radiation dose, and the dose fractionation. Other factors include chemotherapy, the number of fractions, aging, smoking, reduced lung function, and pulmonary hypertension^(1,5)^.

**Figure 1:**
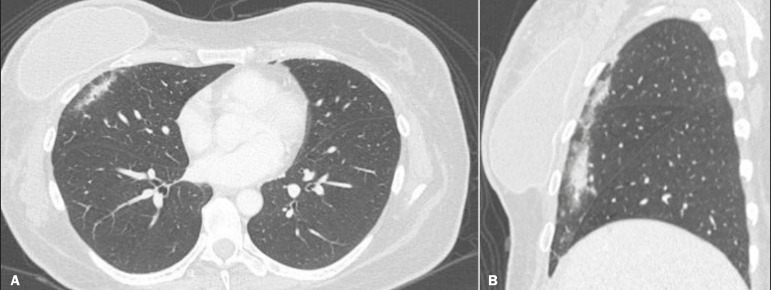
Axial and sagittal CT slices (**A** and **B**, respectively) showing peripheral ground-glass opacities in the region of the irradiated site in a patient who had undergone four weeks of radiotherapy of the right breast for the treatment of breast cancer.

**Figure 2:**
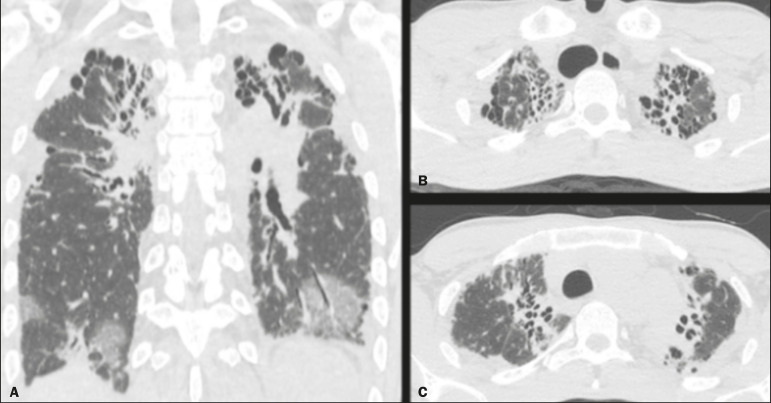
Coronal CT slice (**A**) and axial CT slices (**B,C**), showing signs of architectural distortion, characterized by volume reduction and retractile opacities, together with bilateral cylindrical and varicose bronchiectasis, predominantly in the upper lung fields, in a patient who had undergone chemotherapy and radiotherapy 20 years prior for the treatment of lymphoma.

The computed tomography (CT) findings in the acute phase (Figure 1) are usually heterogeneous ground-glass opacities or areas of consolidation, which are typically located at the radiation site (the capriciously geometric pattern of radiation-induced lesions is an important feature that facilitates their correct identification). In the late phase (Figure 2), septal thickening can be observed over those opacities, resulting in a crazy-paving pattern, with traction bronchiectasis, volume loss, and areas of consolidation^(1,5)^.

The changes seen on chest CT can vary depending on the radiation target, such as bilateral paramediastinal changes seen in cases of lymphoma; bilateral apical opacities seen in cases of head and neck or esophageal cancer, and opacities in the anterolateral regions of the lung in cases of breast cancer^(1)^. In rare cases, radiation-induced lung injury can manifest as organizing pneumonia, which is believed to be immunologically mediated and usually occurs between 6 weeks and 10 months after radiotherapy, being a diagnosis of exclusion^(1,2)^.

**Figure 3:**
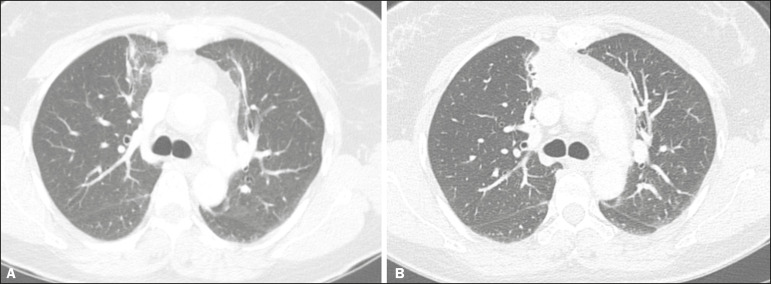
CT scan of a patient with a history of thymoma (**A**), showing ground-glass opacities in the paramediastinal regions, especially on the right. Follow-up CT scan, acquired at 8 months after radiotherapy (**B**), showing a predominance of retractile opacities and volume reduction, suggesting that the radiation-induced lesion had evolved to the fibrotic phase.

**Figure 4:**
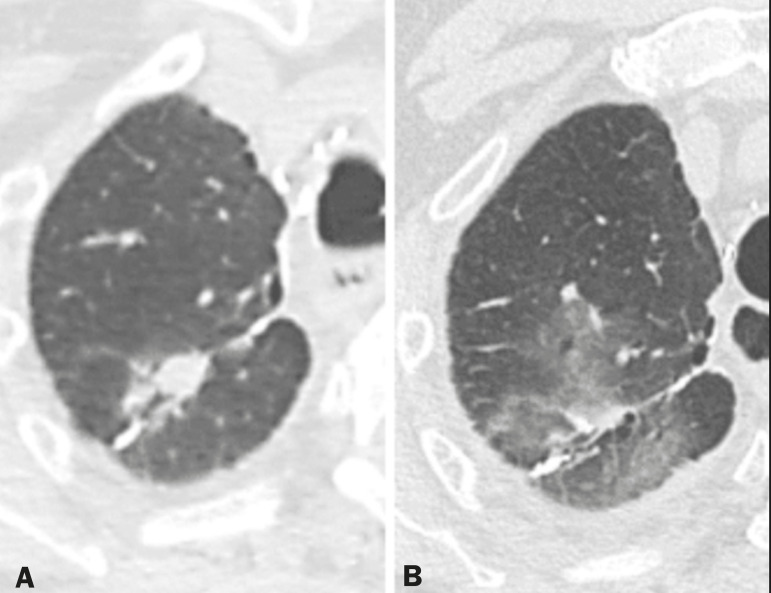
**A:** CT scan of a patient with a recurrent pulmonary nodule. **B:** Follow-up CT scan, acquired at 6 months after stereotactic radiotherapy, showing that the patient had developed a linear atelectatic opacity, with a scarring pattern and adjacent ground-glass opacities, at the radiotherapy site.

**Figure 5:**
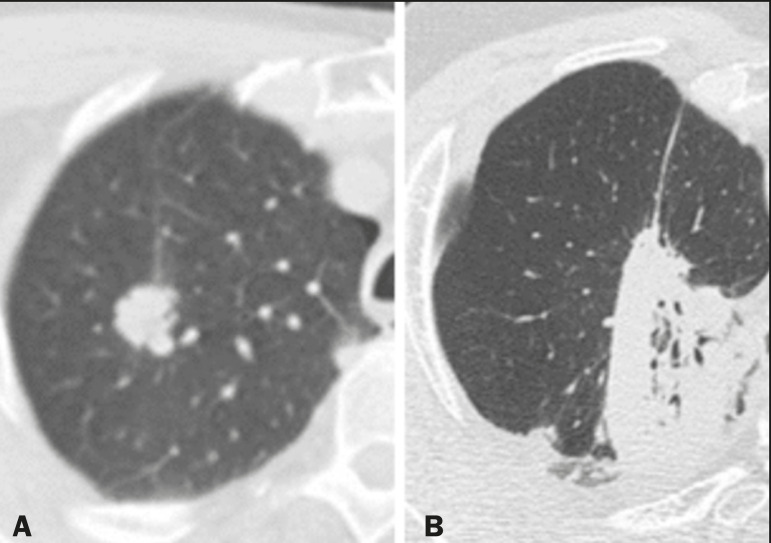
**A:** CT scan of a patient with an irregularly shaped pulmonary nodule at the right apex, which was diagnosed as pulmonary adenocarcinoma. **B:** On a follow-up CT scan, acquired at 9 months after stereotactic radiotherapy, the nodule is no longer well-defined, having been replaced by an area of consolidation, with some air bronchograms and distortion of the local pulmonary architecture, findings characteristic of the mass pattern.

With the use of the most recently developed radiotherapy options, such as conformational, modulated intensity, and stereotactic radiotherapy, different forms of pulmonary changes have been seen. One form resembles that seen after the use of conventional radiotherapy and is known as the modified conventional pattern (Figure 3), other forms including the scarring pattern (Figure 4), which is characterized by band-like opacities, and the mass pattern (Figure 5), which is characterized by an opacity with or without air bronchogram^(1,5)^.

**Figure 6:**
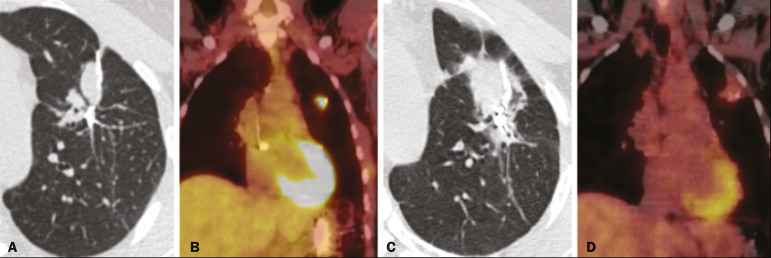
Lung metastasis from an adrenal carcinoma, shown in a CT scan (**A**), as well as in an 18F-FDG PET/CT scan (**B**), on which the standardized uptake value (SUV) was 6.1. **C,D:** Follow-up images acquired at 15 months after treatment with stereotactic radiotherapy, showing that the patient had developed mass-type radiation-induced fibrosis (CT scan in **C**), with an SUV < 5 (18F-FDG PET/CT scan in **D**).

Attention should be paid to glycolytic metabolism in radiation-induced pulmonary changes. It is ideal to wait approximately 6 months after the completion of radiotherapy to perform ^18^F-fluorodeoxyglucose positron-emission tomography/CT (^18^F-FDG PET/CT), as depicted in Figure 6, to avoid false-positive results^(1,4)^.

## PLEURA

Pleural effusions are usually seen within 6 months after the end of radiotherapy. Pleural thickening may be a late effect of radiotherapy and may also show uptake on ^18^F-FDG PET/CT. However, the appearance of or an increase of pleural effusion more than 6 months after the end of radiotherapy should raise suspicion of recurrence, as should the development of nodular thickening^(1,5,6)^.

## HEART

The cardiac effects of radiotherapy can be indirect, such as the development of pulmonary hypertension or dilation of the right ventricle, or direct, such as damage to the coronary arteries, valves, pericardium or myocardium, and all such effects (indirect and direct) usually develop years after radiotherapy^(1)^. Radiotherapy can directly affect the myocardium, inducing myocardial fibrosis, which usually manifests as restrictive cardiomyopathy. In relation to the coronary arteries, stenosis generally affects the proximal portions, with a risk of ischemic heart disease. Periodic evaluation for 5-10 years after radiotherapy is indicated, especially in young patients, if the total radiation dose in the chest was 30-40 Gy^(1,2,7)^. Valve diseases resulting from radiotherapy range from valve thickening and fibrosis to calcification, involvement of the aortic and mitral valves being the most common^(1,7)^.

## PERICARDIUM

**Figure 7:**
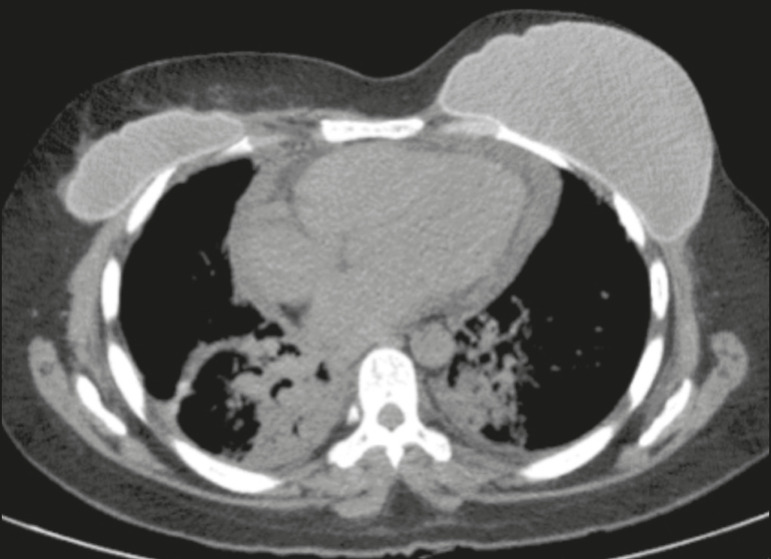
Unenhanced axial CT slice showing moderate pericardial effusion in a patient who had undergone radiotherapy of the left breast for the treatment of breast cancer.

The effects of radiotherapy on the pericardium can be acute or chronic. Pericardial effusion is the most common finding of acute pericarditis (Figure 7), which occurs weeks after radiotherapy and correlates positively with the size of the irradiated field and the radiation dose. In some cases, the condition progresses to chronic pericarditis with pericardial effusion, tamponade, or constriction, the most worrisome complication being constrictive pericarditis. Pericardial thickening can be identified through the use of CT or magnetic resonance imaging (MRI). For the diagnosis of constrictive pericarditis, CT characterizes calcifications more precisely, whereas MRI can demonstrate pericardial thickening, systemic venous dilation, septal rectification, as well as being able to show paradoxical movement of the interventricular septum in cine sequences^(1,2,7)^.

## GREAT VESSELS

Radiotherapy can have effects on the great vessels. Any vessel included in the radiation field can be affected, and radiation-induced injury can be characterized by endothelial dysfunction, vasa vasorum injury, or atherosclerosis. One typical finding of radiation-induced vasculitis is stenosis. Chronic findings include calcifications, vascular occlusion, and pseudoaneurysms^(1)^.

## ESOPHAGUS

**Figure 8:**
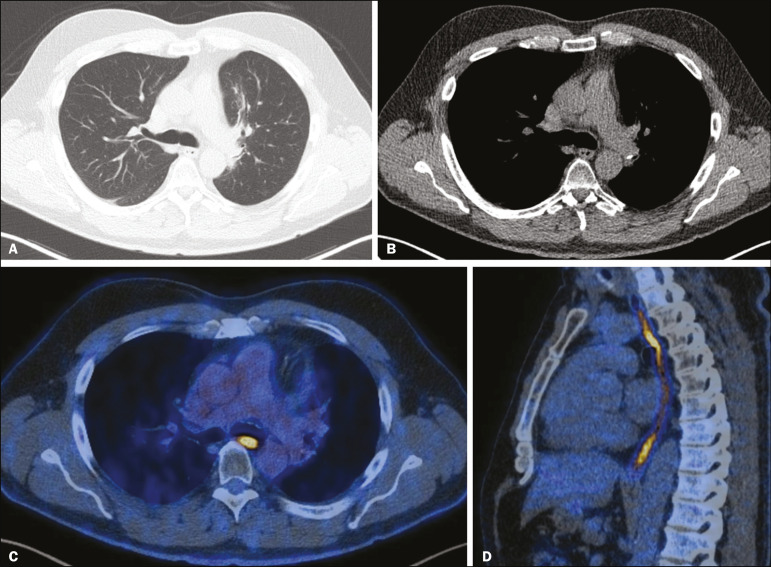
CT scans of a patient who had undergone radiotherapy for the treatment of lung cancer, showing retractile, ground-glass opacities in the left perihilar region (in **A**) and slight thickening of the esophageal walls (in **B**). Axial and sagittal 18F-FDG PET/CT slices (**C** and **D**, respectively), showing increased metabolic activity in the esophagus, suggestive of a radiation-induced inflammatory process (esophagitis).

The risk of developing esophageal complications after radiotherapy depends on the radiation dose, the radiation method employed, and the chemotherapy treatment applied. Adverse effects can be classified as acute or chronic. The acute effects, which occur during or immediately after treatment, and usually disappear within 4-6 weeks, are esophageal dysmotility and esophagitis. Esophagitis manifests as symmetrical thickening of the esophageal wall along its entire length (Figure 8). The late effects occur months to years after irradiation; stenosis is uncommon but can occur 3-18 months after radiotherapy, potentially progressing to chronic ulceration, perforation, or fistula^(1,2)^.

## BREAST

In the acute phase of radiation-induced changes in the breast, there can be diffuse skin thickening. Fatty necrosis and dystrophic calcifications may also be seen. Skin retraction and volume reduction may occur late after the treatment^(1)^.

## BONE

The degree of radiation-induced bone damage is dependent on patient age, the radiation dose administered, and the volume of irradiated tissue. The main bones affected are the ribs, clavicle, humerus, and scapula. First, there is edema, characterized by an increase in signal intensity on T2-weighted MRI scans. At 2-8 weeks after radiotherapy, the bone marrow is replaced by fat, areas of high signal intensity being seen on T1-weighted MRI scans. Late changes are observed at 12 months after radiotherapy, mainly osteoporosis, which increases the risk of fractures, and osteoradionecrosis, which is characterized by areas of heterogeneous bone density, being differentiated from tumors by the presence of multiple lesions in the irradiated field, the absence of a periosteal reaction, and stability over time^(1,6)^.

## THYMUS

One of the complications of radiotherapy is the formation of thymic cysts. On CT scans, such cysts appear as rounded, homogeneous, hypodense images^(1,2)^.

## LYMPH NODES

Calcifications can appear in lymph nodes after radiotherapy. Such calcifications usually appear at approximately 12 months after the treatment and can become increasingly dense over the years^(1,2)^.

## CONCLUSION

Despite the development of various new radiotherapy modalities, there are still side effects due to the proximity of adjacent structures. The radiologist needs to understand such changes in order to interpret the findings appropriately, as well as to identify the complications and recurrences that cause changes in the management of the affected patients.
